# Comprehensive prognostic modeling of locoregional recurrence after radiotherapy for patients with locoregionally advanced hypopharyngeal squamous cell carcinoma

**DOI:** 10.3389/fonc.2023.1129918

**Published:** 2023-03-21

**Authors:** Hongjia Liu, Dan Zhao, Yuliang Huang, Chenguang Li, Zhengkun Dong, Hongbo Tian, Yijie Sun, Yanye Lu, Chen Chen, Hao Wu, Yibao Zhang

**Affiliations:** ^1^ Institute of Medical Technology, Peking University Health Science Center, Beijing, China; ^2^ Key Laboratory of Carcinogenesis and Translational Research (Ministry of Education/Beijing), Department of Radiation Oncology, Peking University Cancer Hospital & Institute, Beijing, China; ^3^ Centre for Medical Image Computing, Department of Medical Physics and Biomedical Engineering, University College London, London, United Kingdom; ^4^ School of Basic Medical Sciences, Peking University Health Science Center, Beijing, China; ^5^ School of Electronics Engineering and Computer Science, Peking University, Beijing, China

**Keywords:** hypopharyngeal squamous cell carcinoma, radiomics, dosiomics, Cox regression, locoregional recurrence

## Abstract

**Purpose:**

To propose and evaluate a comprehensive modeling approach combing radiomics, dosiomics and clinical components, for more accurate prediction of locoregional recurrence risk after radiotherapy for patients with locoregionally advanced HPSCC.

**Materials and methods:**

Clinical data of 77 HPSCC patients were retrospectively investigated, whose median follow-up duration was 23.27 (4.83-81.40) months. From the planning CT and dose distribution, 1321 radiomics and dosiomics features were extracted respectively from planning gross tumor volume (PGTV) region each patient. After stability test, feature dimension was further reduced by Principal Component Analysis (PCA), yielding Radiomic and Dosiomic Principal Components (RPCs and DPCs) respectively. Multiple Cox regression models were constructed using various combinations of RPC, DPC and clinical variables as the predictors. Akaike information criterion (AIC) and C-index were used to evaluate the performance of Cox regression models.

**Results:**

PCA was performed on 338 radiomic and 873 dosiomic features that were tested as stable (ICC_1_ > 0.7 and ICC_2_ > 0.95), yielding 5 RPCs and DPCs respectively. Three comprehensive features (RPC0, P<0.01, DPC0, P<0.01 and DPC3, P<0.05) were found to be significant in the individual Radiomic or Dosiomic Cox regression models. The model combining the above features and clinical variable (total stage IVB) provided best risk stratification of locoregional recurrence (C-index, 0.815; 95%CI, 0.770-0.859) and prevailing balance between predictive accuracy and complexity (AIC, 143.65) than any other investigated models using either single factors or two combined components.

**Conclusion:**

This study provided quantitative tools and additional evidence for the personalized treatment selection and protocol optimization for HPSCC, a relatively rare cancer. By combining complementary information from radiomics, dosiomics, and clinical variables, the proposed comprehensive model provided more accurate prediction of locoregional recurrence risk after radiotherapy.

## 1 Introduction

Compared with invasive surgery, organ-preserving options such as definitive radiotherapy (RT) are more commonly used for patients with previously untreated, newly diagnosed locoregionally advanced hypopharyngeal squamous carcinoma (HPSCC), achieving comparable if not better long-term survival (1–3). However, due to advanced-stage disease, poor performance status, comorbidities, alcohol abuse, and nutritional problems, the 5-year survival rates of patients with HPSCC were only no greater than 40% as reported in various studies (4, 5). Furthermore, the prognosis was also poor, ascribable to the tumor heterogeneity and large outcome uncertainties after standard treatment (6). To improve the prognosis, personalized risk prediction is clinically desirable to support patient-specific selection and optimization of treatment protocols for better tumor control (7, 8), however, existing clinical experience is very subjective and unstable based on conventional parameters such as smoking, drinking, T stage, and lymph node metastasis etc. (9).

By analyzing high dimensional image features that are invisible to radiologists based on multi-modality images such as MRI (10), PET-CT (11) and CT (12), Radiomics has been demonstrated as a promising approach to stratify patients of various risks due to tumor heterogeneity. Unlike the dominant dependence of chemotherapy outcome on the biological varieties as represented by Radiomics features (12), the radiation dose is another key determinative factor of treatment effectiveness for HPSCC patients receiving radiotherapy. The radiation dose distribution is optimized based on patient-specific characteristics such as the shape, volume and location of the tumor and adjacent organs. However, conventional dose-volume-histograms (DVHs) provided limited dosimetric statistics without spatial information, and the predictive accuracy is unsatisfactory (13). Using similar methods as that of Radiomics, Dosiomics analyzes the spatial features of the 3-dimentional patient-specific dose distribution, hence provides better prediction of radiation-induced results (13, 14).

Considering the therapeutic responses of HPSCC patients treated with radiotherapy depend on both tumor heterogeneity and dosimetric variables, this work hypothesized that combined signatures using both Radiomics and Dosiomics features may have more robust statistical correlation with locoregional recurrence of patients with locoregionally advanced HPSCC treated with radiotherapy, potentially supporting more quantitative and personalized clinical decision making such as strategy selection and protocol optimization, which has not been reported in the literature before and is the purpose of this study.

## 2 Materials and methods

### 2.1 Patients

The data of 77 patients pathologically confirmed with HPSCC at our center between October 2011 and July 2020 were retrospectively investigated. The inclusion criteria were (1): lesion located at hypopharynx and pathologically diagnosed squamous cell carcinoma ; (2) administration and completion of laryngeal-preservation treatments: induction chemotherapy (IC)+radical RT or IC+chemoradiation; (3) no postpone, intervention or discontinuation during RT. Data with violation were excluded. All patients underwent disease staging using the American Joint Committee on Cancer Staging System, Eighth Edition (15).

### 2.2 Image acquisition and target delineation

All patients were immobilized in the supine position using thermoplastic head-neck-shoulder masks. Planning CT with intravenous contrast was acquired for structure delineation on a SIEMENS Sensation Open CT scanner using the following protocols: tube voltage 120kV; tube current 320mAs; reconstruction thickness 3 mm; matrix 512 × 512. Target and organs-at-risk (OARs) delineation was manually performed by experienced radiation oncologists according to NCCN and RTOG guidelines respectively (16, 17). All delineations were double checked and approved by senior radiation oncologists per our clinical protocols.

Intensity-modulated radiation therapy (IMRT) or volumetric modulated arc therapy (VMAT) plans were optimized to deliver a prescription dose of 60 Gy to at least 95% of the planning target volume (PTV) in 33 fractions. The dose limitations to OARs were in accordance with RTOG 0615 protocols (18). Simultaneous-integrated-boost (SIB) technique was used to deliver 70 Gy to at least 95% of the planning gross tumor volume (PGTV) in the same plan. All patients were treated with one fraction daily, 5 days per week.

### 2.3 Follow-up protocol and definition of failures

The follow-up was performed at 1 month after the completion of RT, every 3 months during the first 2 years, every 6 months between the third and fifth year, and annually thereafter. At the time of the last follow-up assessment, failure patterns were classified as local, regional, or distant respectively. Local failure was defined as failure of the primary tumor to the treatment. Regional failure was defined as the recurrence in the regional lymph nodes. Distant failure was defined as the appearance of a tumor at any site representing hematogenous dissemination.

### 2.4 Extraction and selection of radiomic and dosiomic features

**Figure 1:**
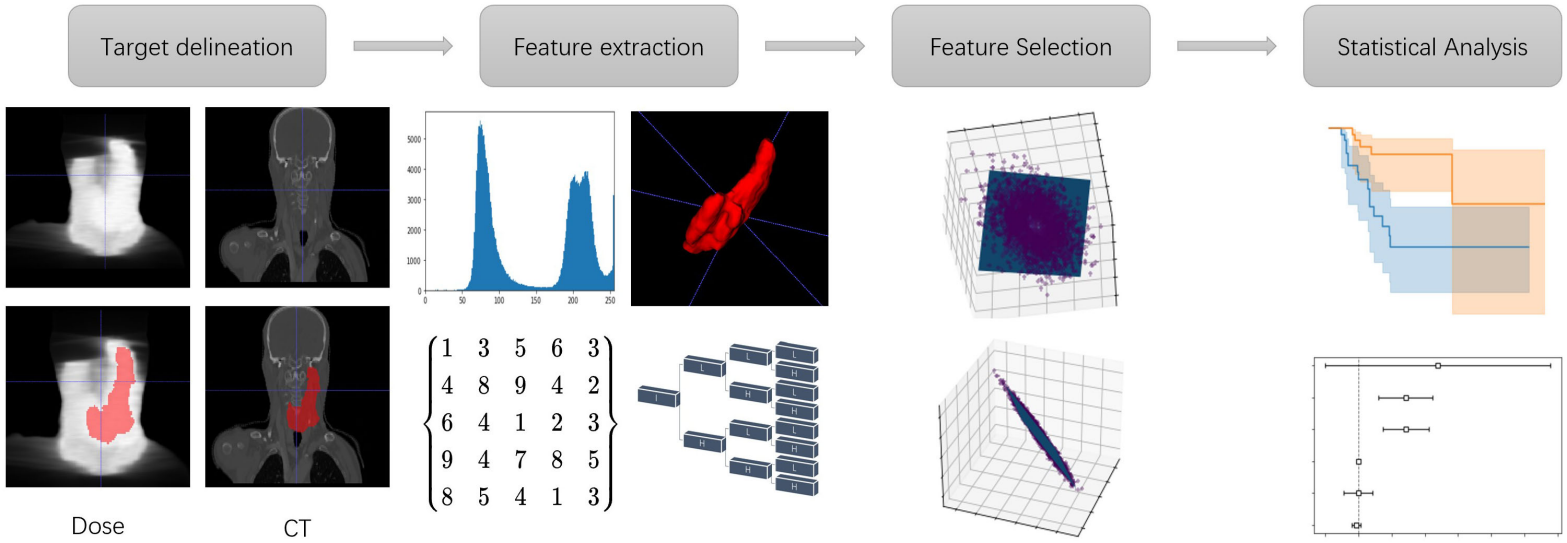
The flowchart of building the statistical analysis model.

The flowchart of building the statistical analysis model is shown in **Figure 1** Using PGTV as the volume-of-interest (VOI), radiomic and dosiomic features were extracted from the planning CT images and dose maps respectively using the third-party python library (19) (https://pyradiomics.readthedocs.io). Basic radiomic features included 18 first-order features, 14 shape-and-volume features and 75 texture features. To expand the feature pool, Log of Gaussian filter and wavelet transform were applied on the planning CT images respectively. The aforementioned texture features were then recalculated, generating 1209 more features. In this study, BiorSplines (bior6.8) was used as the main wavelet function (20). Dosiomic features were extracted using the same procedures from the dose maps.

Consistent with other studies (21, 22), this study evaluated the stability of the extracted radiomic features by applying Guassian noise and random ROI boundary perturbance to the original CT images and VOI boundaries respectively. Three levels of standard variations of the Gaussian noise and two levels of the boundary perturbance distances were used, i.e., 10, 50, 100 HU and 0.5, 1 pixel pitch unit respectively. The intraclass correlation coefficient (ICC) (23) was used as an indicator of feature stability between different groups, which was calculated on the original and the counterpart images respectively, using the definitions of ICC (1,1) ( $ICC_{1}$ ) and ICC (2,1) ( $ICC_{2}$ ) respectively. $ICC_{1}$ was used to indicate interrater reliability of the features and $ICC_{2}$ was used to indicate non-independence of the features respectively (24). $ICC_{1}$ and $ICC_{2}$ were calculated as Eq. (1) and Eq. (2):


(1) $$ICC_{1}=\frac{MSR-MSW}{MSR+\left(k+1\right)*MSW}\;$$

(2) $$ICC_{2}=\frac{MSR-MSW}{MSR}$$

MSR means mean square for rows, MSW means mean square for residual sources of variance and k means the number of raters respectively.

Consistent with the literature, features with $ICC_{1}$ > 0.7 and $ICC_{2}$ > 0.95 were considered as reproducible (25) in the existence of image noise and segmentation errors respectively. As the main influential factor of dosiomic feature robustness, only perturbation of ROI boundary was used to assess the stability of dosiomic features in this study, in accordance with Francesco’s method (26).

Amongst the screened stable features, the most clinically relevant features were further selected using univariate analysis, according to their capability of stratifying the patients into high and low locoregional recurrence risk rates. Log-rank test (27) was used to examine the statistical significance of the inter-group differences between the survival curves of the two groups. Only significant features with p-values<0.05 (statistically significant) were used for further analysis.

### 2.5 Modeling and statistical analysis

Dimensionality reduction of the feature space was performed using Principal Component Analysis (PCA). The first five principal components of radiomic and dosiomic features were used as independent variables of two multivariate Cox regression models respectively, noted as the Radiomic Principal Component (RPC) Model and Dosiomic Principal Component (DPC) Model respectively. A multivariate Cox regression model was also constructed on conventional clinical parameters, noted as the Clinical Model. Kaplan-Meier survival analysis and the log-rank test were used to evaluate the predictive capability of each significant Principal Component and clinical variable respectively.

In addition, the principal components with significant hazard ratio (p< 0.05) were combined with clinical parameters to construct another three comprehensive multivariate Cox regression models, named as Radiomics+Clinical Model, Dosiomics+Clinical Model, and Radiomics+Dosiomics+Clinical Model respectively. The performance of these comprehensive models were evaluated with the partial Akaike information criterion (AIC) (28) and concordance index (C-index) (29) respectively. Defined as $\text{A}IC=2k-2ln\left(\hat{L}\right)$ , AIC was proposed to balance the tradeoff between the ability to fit and the simplicity of the model (30), where k means the number of estimated parameters and $\hat{L}$ means the likelihood function which was maximum. Lower AIC values indicate better models with less complexity. The C-index was used to evaluate discriminative performance of each model. Defined as Eq. (3):


(3) $$C-index=\frac{\sum_{i,j}1_{T_{j}<T_{i}}\cdot 1_{\eta_{j}>\eta_{i}\cdot \delta_{j}}}{\sum_{i,j}1_{T_{j}<T_{i}}\cdot \delta_{j}}$$

C-index (31) presents the proportion of concordant data pairs, where (i,j) is a pair of event; $T_{n}$ means event n’s observation time; ${\text{η}}_{n}$ means event n’s risk; $1_{Tj<Ti}\cdot 1_{\eta_{j}>\eta_{i}}$ means when event j’s risk is higher than event i, event j’s observation time is earlier than event i; ${\text{δ}}_{j}$ means event j happened in $T_{j}$ . As a predictive marker and a time-to-event response variable, C-Index values closer to 1 suggest better model performance.

The statistical analysis was conducted using the following packages of R and Python respectively: the survival package (R) and the lifeline package (Python) were used to execute Kaplan–Meier analysis, build the Cox proportional risk models, and calculate the C-index respectively. Local regional recurrence-free survival (LRRFS), metastasis-free survival (MFS), progression-free survival (PFS), and overall survival (OS) rates were estimated using the Kaplan–Meier method and measured from the first day of treatment to the date of the event. All statistical tests were two-sided, and p values< 0.05 were considered as significant.

## 3 Results

### 3.1 Patient demography and treatment outcomes

The characteristics of the 77 patients included in this study are presented in **Table 1**.

**Table 1:** Clinical characteristics of the 77 patients with locoregionally advanced HPSCC involved in this study.

Characteristics	Number of Patients (%)
Gender	
Male	71 (92.2)
Female	6 (7.8)
Age (years old)	
≥58	47 (61.0)
<58	30 (39.0)
Peripheral invasion^*^	
Yes	55(71.4)
No	22 (28.6)
Total stage (AJCC eighth edition)	
II/III/IVA	55 (71.4)
IVB	22 (28.6)

AJCC, American Joint Committee on Cancer; HPSCC, hypopharyngeal squamous cell carcinoma. *Peripheral invasion: tumor invaded structures surrounding hypopharynx, such as larynx, trachea, oropharynx, and esophagus, et al.

The median follow-up duration was 23.27 (range, 4.83–81.40) months. During the study period, 29 patients experienced disease progression, and 30 patients died due to tumor progression (19), local hypopharyngeal haemorrhage (4), infection (5), car accident (1) and secondary primary cancer (1), respectively. The median PFS and OS estimates were 47.07 months and 36.07 months, respectively. Three-year LRRFS, MFS, PFS, and OS rates were 70.6%, 81.7%, 58.3%, and 48.9%, respectively. Of the 29 patients experienced treatment failure at their last follow-up visit, 13, 2, 9, 3, 0, 1 and 1 patients presented with local only, regional only, distant only, local-regional, regional-distant, local-distant, and local-regional-distant failure, respectively.

### 3.2 Statistical analysis and model evaluation

For each patient, 1321 radiomic features and 1321 dosiomic features were extracted from the planning CT images and dose distributions respectively, from which 338 radiomic features and 873 dosiomic features were identified as stable and reproducible ( $ICC_{1}$ >0.7, $ICC_{2}$ >0.95). After PCA, 5 radiomic principal components and 5 dosiomic principal components were obtained respectively. **Table 2** shows the hazard ratio (HR) and p-values of each principal component and the AIC of Cox regression, for the Clinical Model, Radiomic Principal Components (RPC) Model and Dosiomic Principal Components (DPC) Model respectively. One clinical variable, one RPC and two DPC had significant hazard ratios in Cox regression (p-value< 0.05) respectively.

**Table 2:** Multivariate Cox Regression Analysis for the Clinical Model, Radiomic Principal Components (RPC) Model and Dosiomic Principal Components (DPC) Model respectively.

	HR (95% CI)	z	*P* value
Clinical Model (AIC, 158.61; C-index, 0.663; 95% CI, 0.600–0.725)			
Gender	0.68 (0.20-2.35)	-0.61	0.54
Age	0.92 (0.85-1.00)	-1.99	*0.05*
Stage	1.09 (0.38-3.16)	0.16	0.87
Peripheral invasion	1.25 (0.44-3.51)	0.42	0.67
RPC Model (AIC, 150.73; C-index, 0.762; 95% CI, 0.708–0.815)			
RPC0	1.10 (1.04-1.17)	3.26	<0.01
RPC1	1.08 (0.98-1.21)	1.49	0.14
RPC2	0.94 (0.83-1.07)	-0.96	0.34
RPC3	0.94 (0.79-1.12)	-0.68	0.49
RPC4	0.78 (0.60-1.03)	-1.78	0.08
DPC Model (AIC, 146.33; C-index, 0.783; 95% CI, 0.734–0.832)			
DPC0	1.08 (1.03-1.14)	3.33	<0.01
DPC1	1.04 (0.92-1.19)	0.64	0.52
DPC2	0.94 (0.82-1.06)	-1.03	0.30
DPC3	0.76 (0.60-0.97)	-2.16	0.03
DPC4	1.07 (0.90-1.28)	0.80	0.42

HR, hazard ratio; z, Wald statistic value, the ratio of each regression coefficient to its standard error; CI, confidence interval; RPC, Radiomic Principal Component; DPC, Dosiomic Principal Component; RPCn, DPCn, The nth RPC and DPC obtained by principal component analysis and ranked by weight respectively.

For the combined models, the results of Multivariate Cox Regression Analysis are presented in **Table 3** for the Radiomic+Clinical Model, Dosiomic+Clinical Model and Radiomic+Dosiomic+Clinical Model respectively.

**Table 3:** Multivariate Cox Regression Analysis for the Radiomic+Clinical Model, Dosiomic+Clinical Model and Radiomic+Dosiomic+Clinical Model respectively.

	HR (95% CI)	z	*P* value
Radiomic+Clinical Model (AIC, 148.46; C-index, 0.783; 95% CI, 0.734–0.832)			
RPC0	1.11 (1.04-1.19)	3.24	<0.01
gender	0.56 (0.16-2.01)	-0.89	0.37
age	0.92 (0.84-1.01)	-1.83	0.07
total_stage_IVB	0.36 (0.11-1.25)	-1.60	0.11
Peripheral invasion	1.69 (0.55-5.14)	0.92	0.36
Dosiomic+Clinical Model (AIC, 146.63; C-index, 0.782; 95% CI, 0.730–0.833)			
DPC0	1.07 (1.03-1.11)	3.19	<0.01
DPC3	0.7 (0.54-0.91)	-2.64	0.01
gender	0.98 (0.24-4.06)	-0.03	0.98
age	0.94 (0.86-1.03)	-1.32	0.19
total_stage_IVB	0.4 (0.11-1.49)	-1.36	0.17
Peripheral invasion	1.83 (0.58-5.75)	1.04	0.30
Radiomic+Dosiomic+Clinical Model (AIC, 143.65; C-index, 0.815; 95% CI, 0.770-0.859)			
RPC0	1.09 (1.01-1.18)	2.15	0.03
DPC0	1.04 (0.99-1.08)	1.47	0.14
DPC3	0.7 (0.53-0.93)	-2.47	0.01
gender	0.62 (0.16-2.41)	-0.69	0.49
age	0.91 (0.82-1.01)	-1.76	0.08
total_stage_IVB	0.24 (0.06-0.97)	-2.01	0.04
Peripheral invasion	2.29 (0.73-7.15)	1.43	0.15

HR, hazard ratio; CI, confidence interval; RPC, Radiomic Principal Component; DPC, Dosiomic Principal Component; RPCn, DPCn, The n^th^ RPC and DPC obtained by principal component analysis and ranked by weight respectively.

**Figure 2:**
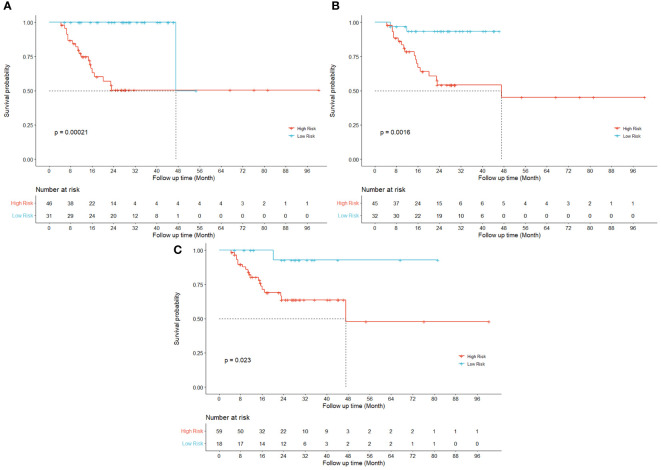
Kaplan-Meier curves for LRRFS. Univariate results are shown for three different principal components in radiomic (**(A)** RPC0) and dosiomic (**(B)** DPC0; **(C)** DPC3) features respectively.

**Figure 2** displays the survival curves of high-risk and low-risk locoregionally advanced patient groups stratified by significant Radiomic and Dosiomic principal components respectively.

**Figure 3:**
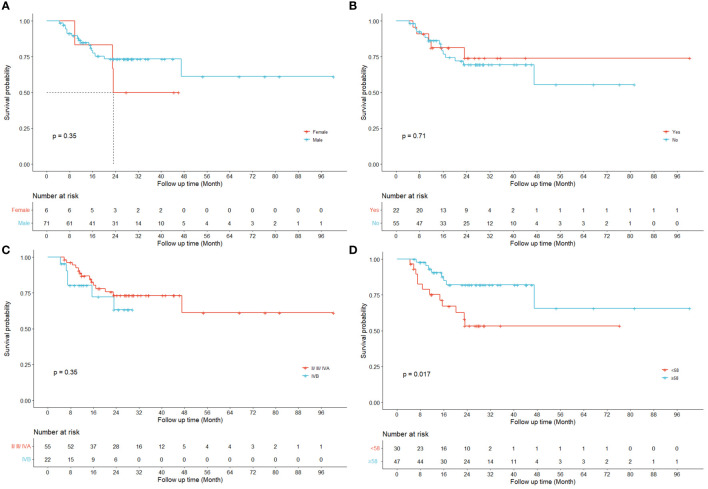
Kaplan-Meier curves for LRRFS. Univariate results are shown for four different clinical variables respectively (**(A)** gender; **(B)** peripheral invasion; **(C)** total stage IVB; **(D)** age).

**Figure 3** displays the survival curves stratified by conventional clinical variables such as gender (a), peripheral invasion (b), total stage (c) and age (d) respectively.

## 4 Discussion

HPSCC is a relatively rare but aggressive malignancy accounting for 5%-10% of head and neck cancer, with high incidence of recurrence and low survival rates (32). There is no sufficient data and evidence to guide precision medicine and prognosis for HPSCC patients. Several clinical prediction models for HPSCC have been published previously (33–35). A reported clinical prediction model for survival in hypopharynx cancer consisted of gender, subsite, TNM classification, Adult Comorbidity Evaluation-27 score (ACE27), body mass index (BMI), hemoglobin, albumin, and leukocyte count. Of these, TNM classification, ACE27, BMI, hemoglobin, and albumin had independent significant associations with survival. But the TNM classification might vary by investigator bias. ACE27 score incorporated 27 ailments which could have mutual effect. Hemoglobin, albumin and leukocyte counts were all baseline data at one time point, and there were 29%, 12% and 2% missing data in the peripheral blood value albumin, leukocyte counts and hemoglobin respectively. All these factors may increase uncertainties of this prediction model (34). The effectiveness and consistency should be further improved in a patient-specific way to assist clinical decision making and treatment protocol optimization.

Previous studies have demonstrated the prognostic values of radiomics features in predicting the risk of HPSCC patients treated with chemoradiation (12, 36) and their key findings were echoed and reconfirmed by our study as shown in the Kaplan-Meier curves for LRRFS (RPC0) in **Figure 2A**. In addition to radiomics, our work also revealed that the principal components of dosiomic features (DPC0 and DPC3) could also stratify patients with different risk (log-rank P<0.05), as shown in **Figures 2B, C**. **Table 2** suggested that dosiomics principal components (C-index=0.783, 95% CI, 0.734–0.832) achieved better predictive performance than that of radiomics (C-index=0.762, 95% CI, 0.708–0.815), if used alone. In contrast, the prognostic performance of conventional clinical variables was less satisfactory (C-index=0.663, 95% CI, 0.600–0.725), consistent with the Kaplan-Meier curves in **Figure 3**. The AIC of RPC Model (150.73) and DPC Model (146.33) were also better than that of Clinical Model (158.61), suggesting better balance was achieved between reducing model regression error and complexity by the former two models.

Compared with the aforementioned single models, the predictive performance of the combined models was improved, as suggested by the lower AIC and higher C-index values shown in **Tables 2**, **3**. Consistent with our hypothesis, the comprehensive model combining the radiomics principal components, dosiomics principal components and conventional clinical variables can best stratify the HPSCC patients with different risks of locoregional recurrence after radiotherapy, as suggested by the largest C-index (0.815) than any other models as shown in **Tables 2** and **3**. This result is also better than that of the previous models using radiomics alone, achieving C-index between 0.690-0.788 (12, 37, 38). Compared with other comprehensive models such as using clinical features + radiomics features by Boot (39) et al (C-index=0.73), and using CT + FDG-PET by Starke (40) et al (C-index=0.80), this work also achieved better reulsts. The comprehensive model also best balanced the ability to fit and the complexity of the model, as suggested by the lowest AIC value (143.65) in **Tables 2**, **3**. We ascribe these improvements to the complementary incorporation of biological heterogeneity as represented by radiomics, personalized treatment intervention as depicted by dosiomics, and empirical evidence as reflected by clinical variables, which were all determinative factors of HPSCC patient outcomes treated with radiotherapy. Firstly, as reported before, the radiomic features are associated with the cancer microenvironment, genetic characteristics, cell growth and histological grading covering the whole tumor area (41–45), providing influential suggestions to the prognosis from the aspects of patient biology. Secondly, as treatment dose distribution was optimized deliberately based on patient-specific anatomies and target prescription, the high-dimensional dosiomic features characterize the personalized specification of treatment intervention. Thirdly, although less robust, clinical variables such as gender, age, stage and peripheral invasion are most familiar to the oncologists, and are broadly used as rule-of-thumb experience to predict the patient prognosis (46–49). The combined data provide complementary information in the quantitative prediction, which is more accurate than that of single factors.

Regarding accessibility, all the required data for the comprehensive modelling, including the planning CT images, dose distribution of treatment plans and conventional clinical variables are readily available for every patient before the start of radiotherapy, potentially facilitating the clinical application of this approach. It provides prompt suggestions before the start of radiotherapy, enabling possible reconsideration of receiving surgery for patients with high risk of local regional recurrence to achieve longer survival than receiving larynx preservation treatments. For patients that have received larynx preservation treatment but are predicted with high risk of local regional recurrence, our model can support personalized clinical suggestions such as more frequent monitoring and examination after radiotherapy.

Regarding methodologies, this work avoided the interference of collinearity to the radiomic and dosiomic features by using PCA. As reported by Traverso et al. (50), the radiomic features have multicollinearity and are largely dependent on tumor volume. Our approach searched for patterns in the data without assuming any a-priori distribution or condition. This study also avoided the bias from subjective selection of cut-off values, by using Youden index for all continuous variables involved in the Kaplan-Meier analysis, consistent with other researches (51, 52).

Although this work is limited by its retrospective design and the relatively small population due to the low morbidity of HPSCC, it provides additional personalized estimation tools and complementary clinical evidence to stratify patients with various risk of locoregional recurrence after radiotherapy, supporting personalized optimization of precision treatment strategies. For instance, if higher locoregional recurrence risks were predicted for a patient receiving function-preserving radiotherapy, more radical treatment such as total laryngectomy might be considered with multi-disciplinary evaluation.

## 5 Conclusions

This study demonstrates that comprehensive models combing radiomic, dosiomic and clinical components displayed better predictive values than any single factor for locoregionally advanced HPSCC treated with chemoradiotherapy, potentially supporting more accurate and prompt clinical decision making such as personalized treatment strategy selection and optimization.

## Data availability statement

The raw data supporting the conclusions of this article will be made available by the authors, without undue reservation.

## Ethics statement

The studies involving human participants were reviewed and approved by Peking University Cancer Hospital (IRB#2019YJZ76). The patients/participants provided their written informed consent to participate in this study.

## Author contributions

HL, DZ and YH contributed equally to this work. HL was Responsible for Statistical Analysis (Email: stevendeliu@stu.pku.edu.cn). All authors contributed to the article and approved the submitted version.
